# The Study of Dried Ginger and Linggan Wuwei Jiangxin Decoction Treatment of Cold Asthma Rats Using GC–MS Based Metabolomics

**DOI:** 10.3389/fphar.2019.00284

**Published:** 2019-04-11

**Authors:** Shan Ran, Fangfang Sun, Yan Song, Xiaoli Wang, Yan Hong, Yanquan Han

**Affiliations:** ^1^The First Affiliated Hospital of Anhui University of Chinese Medicine, Grade 3 Laboratory of TCM Preparation, State Administration of Anhui University of Chinese Medicine, Hefei, China; ^2^Clinical College of Integrated Chinese and Western Medicine, Anhui University of Chinese Medicine, Hefei, China

**Keywords:** dried ginger, Linggan Wuwei Jiangxin decoction, cold asthma, metabolomics, compatibility

## Abstract

Dried ginger is the monarch drug in Linggan Wuwei Jiangxin (LGWWJX) decoction, which is used to treat cold asthma. The purpose of this study was to investigate and compare the effects of dried ginger and LGWWJX decoction for treatment of cold asthma rats at the metabolomics level using gas chromatography–mass spectrometry (GC–MS). OVA and ice water-induced cold asthma were induced in SD rats. The effects of dried ginger and LGWWJX decoction were evaluated by general morphological observation, hematoxylin and eosin staining, inflammatory cell count, IgE, IL-4, IFN-γ quantitation, and visceral index. GC-MS-based metabolomics was performed and analyzed using multivariate statistical analysis. Biomarker identification, pathway analysis, correlations between identified biomarker, and efficacy indices were performed. The results showed that dried ginger and LGWWJX decoction had obvious effects on cold asthma rats. Thirty-seven metabolites (15 in serum and 22 in urine) associated with cold asthma were identified. These metabolites were mainly carbohydrates, fatty acids and their products, organic acids, and others. Seven pathways were identified by MetaboAnalyst 4.0 metabolic pathway analysis. After intervention with dried ginger and LGWWJX decoction, the majority of altered metabolites and metabolic pathways returned to control levels. LGWWJX decoction regulated more metabolites of carbohydrates and fatty acids, which contribute to energy metabolism and oxidative stress in cold asthma, than dried ginger. We concluded that dried ginger and LGWWJX decoction both were effective for treatment of cold asthma. LGWWJX decoction was more effective than dried ginger for treatment of cold asthma. This study evaluated the effects of dried ginger and LGWWJX decoction on cold asthma at the metabolomics level. It provides a reference for the research on the compatibility of Chinese Medicine.

## Introduction

Ginger is a Chinese medicine (CM) used as a food and as a medicine, and is often used to treat cold syndrome through the property of warming. Dried ginger (Zingiberis Rhizoma, GanJiang, GJ) is processed from fresh ginger and is also commonly used in CM. It is used to treat cold asthma by “warming the stomach to dispel cold, warming the lung to resolve fluid,” and it has significant anti-inflammatory, anticancer, antipyretic, and antioxidant properties (Bak et al., 2012; Townsend et al., 2014). A previous report showed that phenolic compounds in GJ can prevent and treat asthma by inhibiting expression of TH2 and TH1 cells (Khan et al., 2015). However, CM in clinical application is mainly based on CM prescription.

Linggan Wuwei Jiangxin (LGWWJX) prescription originated from the “Jingui Yaolüe” (“Synopsis of Golden Chamber”), written by Zhongjing Zhang, who was one of the most famous Chinese doctors during the Han Dynasty. LGWWJX has been used to treat cold asthma for over a 1000 years. It consists of Zingiberis Rhizoma (Gan Jiang, GJ, monarch drug), Poria (Fu Ling, FL), Glycyrrhizare Radix Et Rhizoma (GanCao, GC), Asari Radix Et Rhizoma (Xi Xin, XX), and Schisandrae Chinensis Fructus (Wu Wei Zi, WWZ). Interestingly, GJ is the main component in LGWWJX, which can also be used to treat cold asthma. Our previous study demonstrated significant differences between various processed ginger products on blood stasis syndrome (Han et al., 2016). In this study, we proposed that there were differences in the compatibility of GJ for treatment of disease.

Metabolomics is a part of biological systems to monitor global small molecule endogenous metabolite changes induced by biochemical reactions under a given set of physiological conditions *in vivo*. It is a useful technique for study of CM. As metabolomics provides a comprehensive assessment of cellular pathways, it has recently attracted much attention in the treatment of diseases with CM (Wang et al., 2005; Shi et al., 2012; Qi et al., 2014). Gas chromatography–mass spectrometry (GC–MS) is a technique used for metabolomics analysis, which is characterized by high sensitivity and numerous available databases. GC–MS has been used extensively in metabolomics studies (Vinaixa et al., 2016). Therefore, a metabolomics study was performed using GC–MS profiling combined with multivariate statistical analysis to monitor biomarkers and altered metabolism in serum and urine in ovalbumin (OVA) and ice water-induced cold asthma rats. We compared the effects of GJ and LGWWJX on cold asthma at the metabolite level. First, differences in metabolomic biomarkers in serum and urine were identified between the control and the treated group. Then, the effects of treatment with GJ and LGWWJX on cold asthma were evaluated. In addition, the therapeutic mechanisms of GJ and LGWWJX on cold asthma were identified by analyzing metabolic pathways.

## Materials and Methods

### Ethics Statement

Male Sprague Dawley (SD) rats were purchased from the Laboratory Animal Center, Medical University of Anhui Province. All experiments were subject to approval by the Committee on the Ethics of Animal Experiments of Anhui University of Chinese Medicine. All experiments were performed in accordance with the “Regulations for the Administration of Affairs Concerning Experimental Animals” issued by the National Science and Technology Commission.

Rats from all groups were anesthetized by intraperitoneal injection with pentobarbital (50 mg narcoren/kg body weight) and fentanyl (0.05 mg/kg body weight).

### Materials

Ovalbumin (A5253) was purchased from Sigma Aldrich, Co., St. Louis, MO, United States, *N*-methyl-*N*-trimethylsilyl-trifluoro-acetamide (MSTFA, G35006), methoxyamine hydrochloride (90663189), and pyridine (20170104) were obtained from West Asia Chemical Industry, Co., Ltd., Shan Dong. Urease (EK170062) was supplied by Yi Ka, and methanol (20170905) and chloroform (20170321) were purchased from Sinopharm Chemical Reagent, Co., Ltd. (Beijing, China). All other chemicals were of analytical grade and used as received.

All herbs were purchased from the pharmacy of the First Affiliated Hospital of Anhui University of Chinese Medicine, and they were authenticated by Prof. Shoujin Liu (Anhui University of Chinese Medicine, Hefei, China). LGWWJX prescription contains the following five CMs:

(1) Zingiberis Rhizoma (Gan Jiang, GJ), which warms the spleen and stomach for dispelling cold, warming the lungs, and resolving retained fluid.
(2) Poria (Fu Ling, FL), which clears damp and promotes diuresis.
(3) Glycyrrhizare Radix Et Rhizoma (Gan Cao, GC), which helps to relieve coughs and reduce sputum;
(4) Asari Radix Et Rhizoma (Xi Xin, XX), which expels wind-evil and removes cold;
(5) Schisandrae Chinensis Fructus (Wu Wei Zi, WWZ), which eases coughing and tonifies kidney yang and moderates the properties of herbs.

### GJ and LGWWJX Decoction Preparations

Linggan Wuwei Jiangxin decoction was prepared according to the methods in our previous studies. Briefly, 20 times the minimum dose was weighed, which included Gan Jiang (180 g), Fu Ling (240 g), Gan Cao (180 g), Xi Xin (100 g), and Wu Wei Zi (100 g), and soaked in water at a 1:12 (w/v) ratio for 50 min. The water was then heated to boiling and was simmered for 40 min, then filtered. After filtration, the dregs were added to water and the above steps were repeated. The filtrates were then combined and concentrated to 2.2 g/mL. The preparation process for GJ decoction was identical to that of LGWWJX, and included 180 g of Gan Jiang. GJ was concentrated to 0.5 g/mL. GJ and LGWWJX decoctions were stored at 4°C and heated before use. The chemical components of LGWWJX decoction are shown in Supplementary Figure S1 and Supplementary Table S1.

### Animal Model Establishment and Assessment

#### Animal Model Establishment

Rats were randomly divided into five groups (*n* = 8 per group) as follows: control group (C), model groups (M), Gan Jiang group (GJ), LGWWJX treatment group, and positive control (Guilong Kechuanning capsule) group (PC). According to a previous study (Yan, 2016), rats in the M, GJ, LGWWJX, and PC groups were sensitized by intraperitoneal injections of 10% OVA (100 mg OVA, 100 mg aluminum hydroxide) in 0.5 mL of saline on the 1^st^ and 7^th^ days. The C group was injected with an equal volume of saline. On days 15–30, the M, GJ, LGWWJX, and PC groups were challenged with 1% OVA for 30 min, then subjected to a swimming test in ice water (14 ± 2°C) for 30 min. The GJ group received the Gan Jiang decoction (4.86 g/kg) by gavage, and the LGWWJX group received the LGWWJX decoction (21.6 g/kg) by gavage for 30 from the day of challenge (0.5 h after challenge). The PC group was administered Guilong Kechuanning capsule (2.43 g/kg) by gavage. The C and M groups were given distilled water. The doses of LGWWJX decoction and Guilong Kechuanning capsule were converted according to the body surface area ratio between humans and the animals in the study. The doses were sixfold than the corresponding clinical prescription dose for a 60 kg human, which is an effective moderate dose based on preliminary experiments. The dose of dry ginger was consistent with that of the amount of dried ginger in LGWWJX decoction.

#### Model Assessment Indicators

General morphological observation, histopathology analysis of the lungs, inflammatory cell count in bronchoalveolar lavage fluid (BALF), IgE, IL-4, IFN-γ in serum, and visceral index were chosen as model assessment indicators.

### Histopathology Analysis

After obtaining BALF, the upper right middle lung lobe was removed and fixed in 10% neutral buffered formalin. The specimens were dehydrated and embedded in paraffin. Five-micrometer sections of fixed embedded tissues were cut using a Leica model RM2135 rotary microtome (Leica, Nussloch, Germany) and stained with hematoxylin and eosin. Histological analyses were performed by two independent experimenters blinded to the treatment groups.

### Cell Count in BALF and IgE, IL-4, and IFN-γ Detection in Serum

Rats underwent bronchoalveolar lavage with three 1-mL aliquots of ice-cold PBS via an endotracheal cannula. BALF recovery was greater than 70%. BALF was centrifuged at 1,500 rpm for 10 min at 4°C. The supernatant was discarded and the pellet was kept for further analysis. The number of total cells was counted using a hemocytometer, and cytologic classification in the bronchial alveolar lavage fluid (BALF) was determined using Wright Giemsa staining. Enzyme-linked immunosorbent assay (ELISA) (San Diego, CA, United States) was used to determine the expression levels of IgE, IL-4, and IFN-γ in serum according to the manufacturer’s instructions. The absorbance was measured at 450 nm using a microplate reader (RT-6000, Leidu, United States).

### Visceral Index

The hearts, livers, spleens, lungs, and kidneys of the rats were weighed. The visceral index was determined by calculating the ratios of weights of each organ to the corresponding body weights of the animals.

### Serum and Urine Samples Collection

The day before sacrifice, C, M, GJ, and LGWWJX rats were housed in metabolic cages (1 per cage) to collect urine for 24 h. The animals were free to drink water but were fasted. All urine samples were collected and immediately centrifuged at 3,000 rpm for 10 min at 4°C and the supernatant was stored at -80°C. The rats were then anesthetized by intraperitoneal injection of pentobarbital (50 mg narcoren/kg body weight) and fentanyl (0.05 mg/kg body weight) to obtain blood from the abdominal aorta. Blood samples were incubated for 3 h at room temperature, centrifuged at 3,000 rpm for 10 min, and the supernatants were collected and stored at -80°C.

### Sample Preparation

#### Serum Sample Preparation

Serum metabolites were analyzed following chemical derivatization according to a previously published procedure with minor modifications (Papadimitropoulos et al., 2018). One hundred microliters of thawed serum sample was added to 1.5 mL microcentrifuge tubes, followed by addition of 350 μL of methanol. The samples were vortexed for 10 s, then centrifuged at 12,000 rpm for 10 min at 4°C. A 350 μL aliquot of the supernatant was transferred into a 2 ml GC/MS glass vial and dried under nitrogen at room temperature. The residue was derivatized using a two-step procedure. First, 80 μL of methoxyamine hydrochloride (20 mg/mL in pyridine) was added to each vial, mixed gently, shaken, and incubated for 2 h at 37°C. Then, 100 μL of BSTFA (1% TMCS, v/v) was added, and the tubes were shaken and incubated for 1 h at 70°C. The derivatized samples were cooled to room temperature prior to GC–MS analysis.

#### Urine Samples Preparation

Urine samples also required chemical derivatization. One hundred microliters of thawed urine was transferred to 1.5 mL eppendorf tubes and 10 μL of urease (80 mg/ml) was added. The samples were then mixed gently, shaken, and incubated for 1 h at 37°C. Next, 350 μL of methanol:chloroform (3:1, v/v) was added and the samples were vortexed for 10 s. The remaining steps were the same as those described in the serum sample derivatization preparation.

### Quality Control Samples Preparation

To monitor repeatability and reliability of the method, quality control samples (QCs) were prepared by mixing equal aliquots from each serum or urine sample. QC samples were then analyzed using the same method as the prepared urine. The QC samples were injected at regular intervals (every eight samples) throughout the analytical run to provide a set of data from which repeatability and reliability could be assessed.

### GC–MS Analysis

Gas chromatography–mass spectrometry analysis was performed using a Bruker 45X gas chromatograph system coupled with a SCION single quadrupole mass spectrometer (Bruker, United States).

Derivatized samples (1 μL each) were injected onto the gas chromatograph system with a split inlet equipped with a DB-5MS capillary column (30.0 μm × 250 μm inner diameter, 0.25 μm film thickness) under the following conditions: initial oven temperature was set at 50°C for 2 min, and increased to 100°C at a rate of 8°C/min, then increased to 120, 180, 200, 250 (alternate between 4 and 8°C/min), and ultimately to 280°C for 3 min. Helium was used as the carrier gas at a flow rate of 1.0 mL/min in constant flow mode and the injector split ratio was set to 20:1. The temperatures of the injector, and ion source and MS were set to 279 and 219°C, respectively. The energy was -70 eV in electron ionization mode. MS data were acquired in full-scan mode across mass-to-charge ratios (m/z) of 50–500.

### Data Processing and Statistical Analysis

All the GC–MS raw files from serum and urine samples were converted to Net. CDF format using proteowizard software. Peak identification, peak alignment, and peak filtration were performed using XCMS^1^ online to obtain a data matrix composed of m/z values, retention times, ion fragments, and peak areas. Peak areas were normalized using Excel, then imported into SIMCA-P 13.0 (Umetrics AB, Umeå, Sweden), in which multivariate statistical analysis was performed. Before analysis, data were mean centered and unit variance scaled. Principal component analysis (PCA) was used to display the overall differences. Partial least-squared discrimination analysis (PLS-DA) and orthogonal projection to latent structure-discriminate analysis (OPLS-DA) were used to verify the model and explore different biomarkers between groups by incorporating known classifications. Then, the variable importance for project values (VIP) exceeding 1 were selected first and these metabolites were subsequently analyzed by paired *t*-test analysis using SPSS 23.0 (International Business Machines, Corp., Armonk, NY, United States), and *p* < 0.05 was considered to be a significant difference. VIP > 1 and *p* < 0.05 were criteria for screening differential metabolites. Metabolite identification was performed by executing similarity searches in the NIST11.L mass spectral library (National Institute of Standards and Technology, Gaithersburg, MD, United States) and metabolites with a NIST match factor of ≥ 700 were investigated. One-way analysis of variance (ANOVA) (SPSS23, International Business Machines, Corp., Armonk, NY, United States) was used to evaluate pharmacodynamics effects among the four groups. All experimental data are presented as mean ± standard deviation.

## Results

### Assessment of Cold Asthma Rats

#### General Morphological Observation

Rats in group C moved freely, had shiny hair, and exhibited smooth breathing. Rats in the M group exhibited slow movement, nose scratching, sneezing, and wheezing, and had swollen claws, white lips, and dark hair.

#### Histopathology Analysis

**FIGURE 1:**
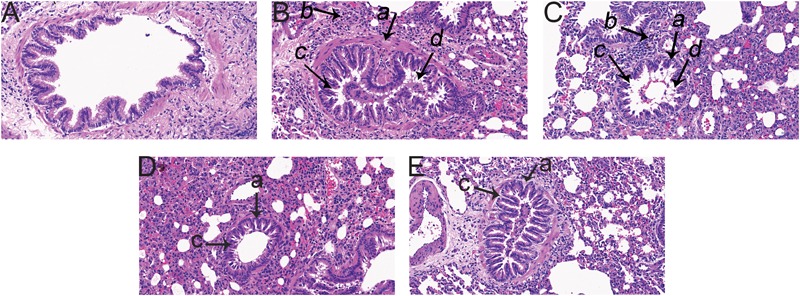
Pathological changes in lung tissue observed by hematoxylin and eosin (HE) staining (Light microscopy, ×200). 4.86 g/kg Gan Jiang and 21.6 g/kg of Linggan Wuwei Jiangxin (LGWWJX) decoction significantly reduced inflammation and pathological changes compared with the model group. LGWWJX decoction alleviated airway wall thickening and inflammatory cell infiltration to a greater extent than GJ. **(A)** control; **(B)** model; **(C)** Gan Jiang (GJ, 4.86 g/kg); **(D)** LGWWJX decoction (LGWWJX, 21.6 g/kg); **(E)** postive control (PC, Guilong Kechuanning, 2.43 g/kg). (a) airway wall; (b) inflammatory cell infiltration; (c) airway luminal narrowing; (d) mucous plug.

Pathological changes in lung tissue were assessed by hematoxylin and eosin (HE) staining of the paraffin embedded sections (Figure 1). Lungs of M group animals exhibited airway wall thickening, airway luminal narrowing, inflammatory cell infiltration around mucosa of bronchioles, and epithelial cell shedding (Figure 1B), which indicated that the model had been successfully established. These changes were markedly ameliorated by GJ and LGWWJX treatments, and LGWWJX exerted a greater effect.

#### Cell Count in BALF and IgE, IL-4, and IFN-γ Detection in Serum

As shown in Table 1, total cells in BALF and the percentage of eosinophils, mononuclear and neutrophils in the M group were dramatically increased compared to that in the C group. These results are summarized in Table 1 and Supplementary Figure S2. IgE, IL-4 were markedly increased, but IFN-γ was significantly decreased, in the M group. These results are summarized in Supplementary Figure S3. These results demonstrated that GJ and LGWWJX treatment could ameliorate inflammation associated with asthma.

**Table 1:** Inflammatory cells and cytokines in the four groups.

Group	Cell count in BALF				Inflammatory cytokines in serum		
	Total cells (× 10^6^)	Eosinophils (%)	Mononuclear (%)	Neutrophil (%)	IgE (μg⋅mL^-1^)	IL-4 (ng⋅mL^-1^)	IFN-γ (ng⋅mL^-1^)
C	2.23 ± 1.27	1.06 ± 0.98	12.50 ± 1.70	11.25 ± 1.39	102.10 ± 3.47	248.70 ± 5.02	1322.00 ± 12.61
M	28.41 ± 3.24^##^	30.44 ± 3.87^##^	62.88 ± 2.13^##^	25.31 ± 3.06^##^	290.40 ± 5.96^##^	927.50 ± 30.63^##^	507.10 ± 16.16^##^
GJ	3.70 ± 1.00^∗∗^	19.81 ± 5.74^∗^	42.73 ± 2.14^∗∗^	18.88 ± 2.12^∗∗^	232.20 ± 12.61^∗∗^	697.20 ± 21.45^∗∗^	1149.0 ± 30.59^∗∗^
LGWWJX	3.49 ± 1.13^∗∗^	9.37 ± 1.21^∗∗^	45.81 ± 2.25^∗∗^	13.87 ± 2.55^∗∗^	188.90 ± 3.45^∗∗^	512.60 ± 21.37^∗∗^	834.01 ± 26.37^∗∗^
PC	2.82 ± 0.30^∗∗^	5.63 ± 0.43^∗∗^	30.13 ± 0.59^∗∗^	13.13 ± 0.91^∗∗^	159.04 ± 2.60^∗∗^	407.10 ± 19.58^∗∗^	1113.20 ± 37.41^∗∗^

*Data are represented as means ± SD. *n* = 8. ^##^Compared with control group, *p* < 0.01. ^∗^Compared with model group, *p* < 0.05. ^∗∗^Compared with model group, *p* < 0.01.*

#### Visceral Index

As shown in Table 2, heart and lung indices were increased in the M group compared with the C group. No other group comparisons resulted in significant differences. Treatment with GJ and LGWWJX decoctions and PC returned heart and lung indices to normal levels.

**Table 2:** Visceral index results.

	Heart index	Liver index	Spleen index	Lung index	Kidney index
C	0.31 ± 0.04	2.82 ± 0.32	0.23 ± 0.06	0.91 ± 0.25	0.59 ± 0.05
M	0.38 ± 0.05^##^	3.11 ± 0.24	0.30 ± 0.08	0.96 ± 0.13^##^	0.63 ± 0.06
GJ	0.35 ± 0.07^∗∗^	2.99 ± 0.35	0.24 ± 0.06	0.92 ± 0.24^∗∗^	0.63 ± 0.09
LGWWJX	0.33 ± 0.05^∗∗^	2.85 ± 1.12	0.22 ± 0.07	0.83 ± 0.22^∗∗^	0.63 ± 0.07
PC	0.31 ± 0.05^∗∗^	2.80 ± 0.33^∗^	0.24 ± 0.07	0.90 ± 0.26^∗∗^	0.61 ± 0.06

*(C) Control group, (M) model group, (GJ) 4.86 g/kg Gan Jiang decoction group, (LGWWJX) 21.6 g/kg Linggan Wuwei Jiangxin decoction group, (PC) 2.43 g/kg positive control group. Data are represented as means ± SD. *n* = 8. ^##^Compared with control group, *p* < 0.01. ^∗^Compared with model group, *p* < 0.05. ^∗∗^Compared with model group, *p* < 0.01.*

### GC–MS Analysis

#### QC Samples Analysis

Use of QC samples confirmed system suitability and analytical stability over the course of analytical data collection. QC data can be used as quantitative indicators of random errors or fluctuations during the analytical run. GC–MS total ion current (TIC) chromatograms of QC samples were captured (Supplementary Figure S4), and the chromatographic peaks in the QC samples nearly all agreed, indicating good stability of the instrument and method.

#### GC–MS Analysis

**FIGURE 2:**
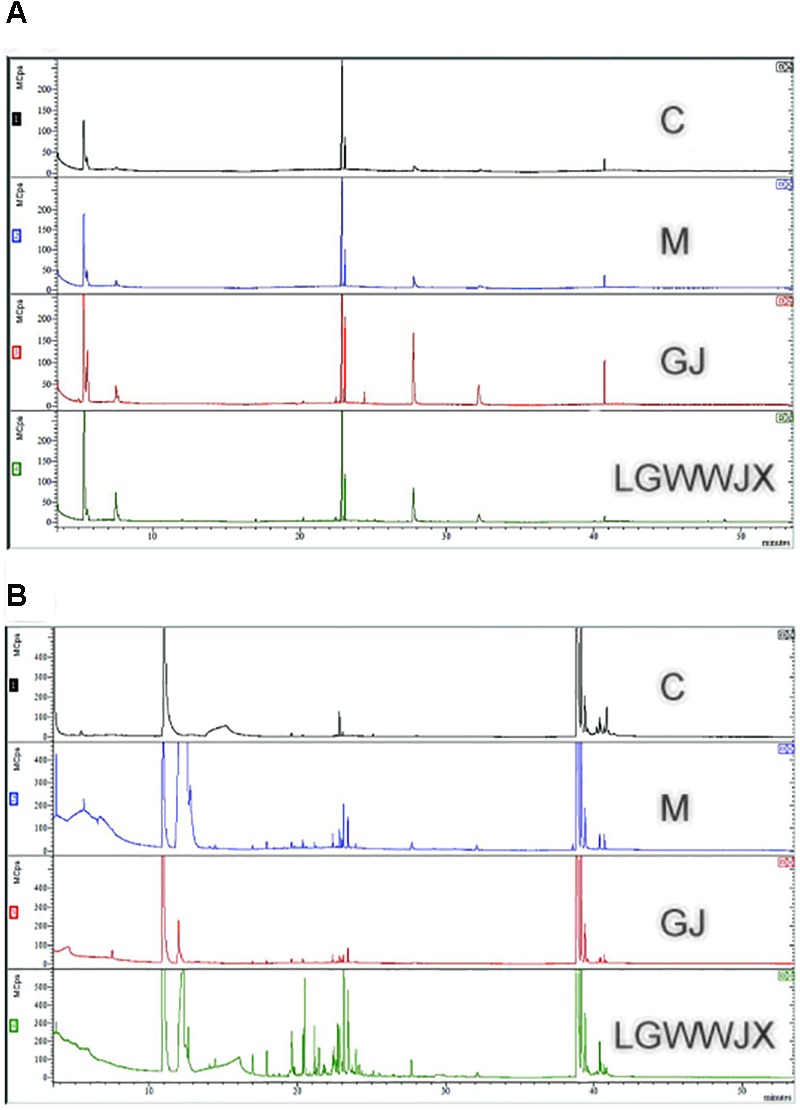
Representative GC–MS TIC chromatograms of control (C), model (M), Gan Jiang (GJ) decoction, and LGWWJX decoction groups in rat serum **(A)** and urine **(B)**.

TIC chromatograms of serum and urine from the C, M, GJ, and LGWWJX groups are shown in Figure 2A,B. However, no clear differences were observed.

### Multivariate Statistical Analysis of Metabolites

**FIGURE 3:**
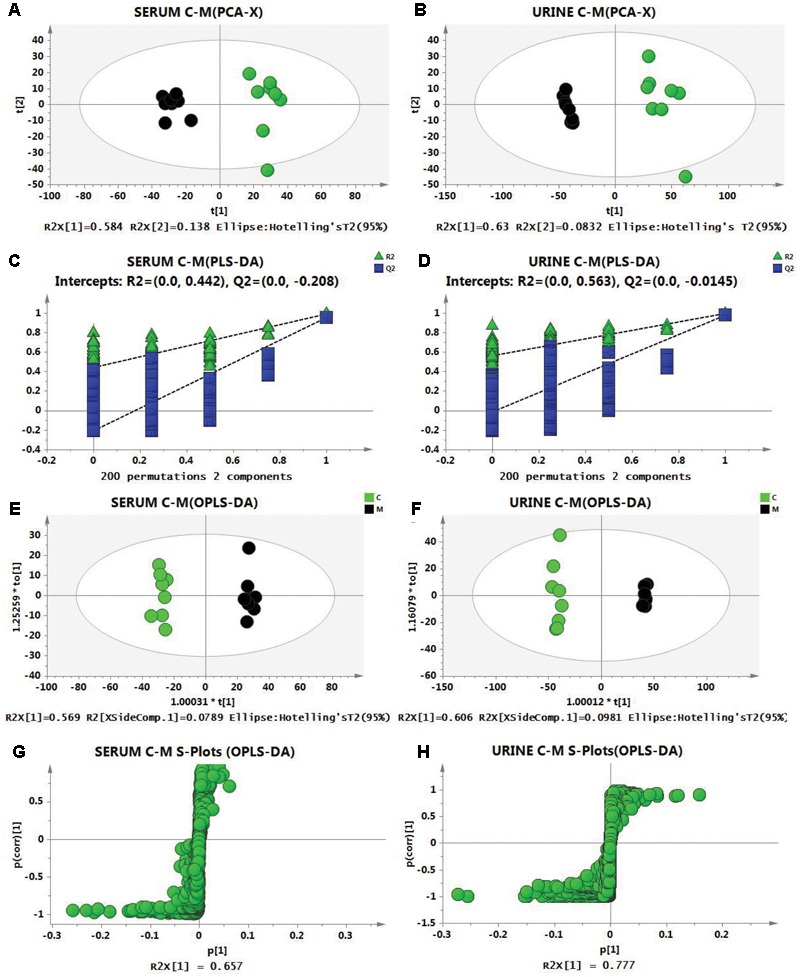
Score plots from the PCA model of the control group (green) vs. the model group (black) for **(A)** serum and **(B)** urine. Two hundred permutations of the PLS-DA model for serum **(C)** and urine **(D)**. R^2^ (green triangle); Q^2^ (blue square). Score plots form the OPLS-DA model of the control group (C, green) vs. the model group (M, black) for serum **(E)** and urine **(F)**. S-Plots from the OPLS-DA model for serum **(G)** and urine **(H)**.

To evaluate metabolic profiles, PCA was performed between the C group and the M group for the serum and urine samples with the following results: *R*^2^ = 0.794 and *Q*^2^ = 0.635 for serum, and *R*^2^ = 0.713 and *Q*^2^ = 0.576 in urine, which indicated that the models were of good quality and provided accurate predictions. The score plots of PCA (Figure 3A,B) showed obvious differences between the C group and the M group, which indicated that the metabolic profiles of cold asthma rats were altered. Then, PLS-DA analysis was performed, and permutation test (permutation number: 200) results were as follows: *R*^2^ = 0.442 and *Q*^2^ = -0.208 in serum, and *R*^2^ = 0.563 and *Q*^2^ = -0.0145 in urine, which demonstrated robustness and good predictive ability of the model (Figure 3C,D). To screen differential metabolites and maximize discriminatory ability of serum and urine metabolites between the groups, orthogonal partial least-squares discriminant analysis (OPLS-DA) was performed. As shown in the score plot (Figure 3E,F), the serum and urine samples in the M group were significantly different from those in the C group. The S-plots (Figure 3G,H) showed differential metabolites between the two groups, and variable importance in the projection (VIP) was obtained based on OPLS-DA with a threshold of 1.0.

**FIGURE 4:**
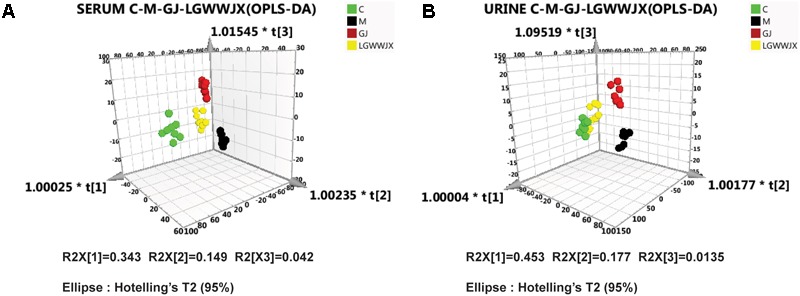
OPLS-DA scores 3D plots. **(A)** Comparison of C (green), M (black), GJ (red), and LGWWJX (yellow) in serum (R^2^X = 0.855, R^2^Y = 0.896, Q^2^ = 0.659). **(B)** Comparison of C (green), M (black), GJ (red), and LGWWJX (yellow) in urine (R^2^X = 0.817, R^2^Y = 0.899, Q^2^ = 0.639).

In addition, we also investigated the differences in metabolic profiles between the M group and the GJ group, and the M group and the LGWWJX group using OPLS-DA analysis. Score 3D plots (Figure 4A,B) from OPLS-DA were used to maximize discrimination of metabolite differences among the four groups. Figure 4 shows that the metabolomic profiles of the GJ and LGWWJX treatment groups had a tendency to return to a profile more similar to the control group, and the metabolomic profile of the LGWWJX group was closest to that of the C group. These results suggested that GJ and LGWWJX both exerted effects on metabolism in cold asthma rats, and LGWWJX exerted more powerful effects than GJ on cold asthma.

### Potential Biomarkers

**FIGURE 5:**
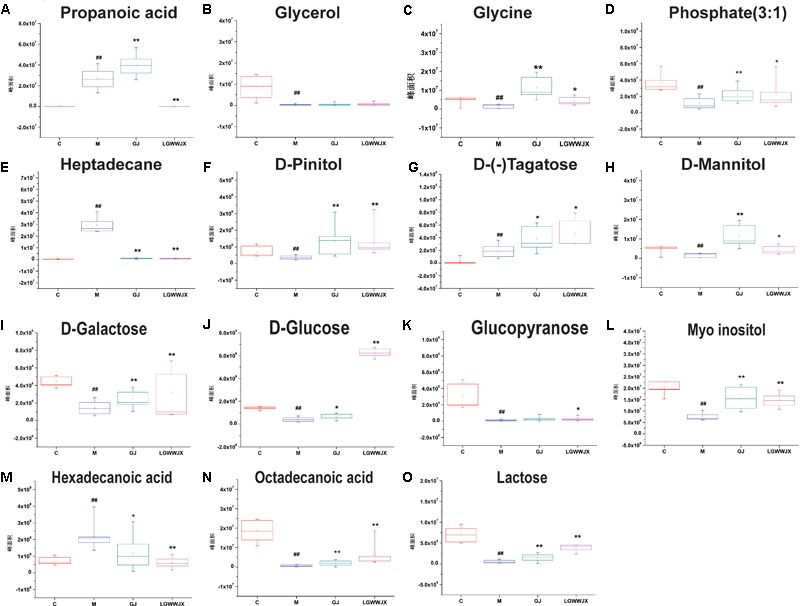
Expression levels of differentially expressed metabolites in serum. **(A)** Propanoic acid; **(B)** Glycerol; **(C)** Glycine; **(D)** Phosphate (3:1); **(E)** Heptadecane; **(F)** D-Pinitol; **(G)** D-(–)Tagatose; **(H)** D-Mannitol; **(I)** D-Galactose; **(J)** D-Glucose; **(K)** Glucopyranose; **(L)** Myoinositol; **(M)** Hexadecanoic acid; **(N)** Octadecanoic acid; **(O)** Lactose. C, control group; M, model group; GJ, GanJiang decoction group; LGWWJX, LGWWJX decoction group. ^#^*p* < 0.05, ^##^*p* < 0.01, compared with the control group. ^∗^*p* < 0.05, ^∗∗^*p* < 0.01, compared with the model group.

**FIGURE 6:**
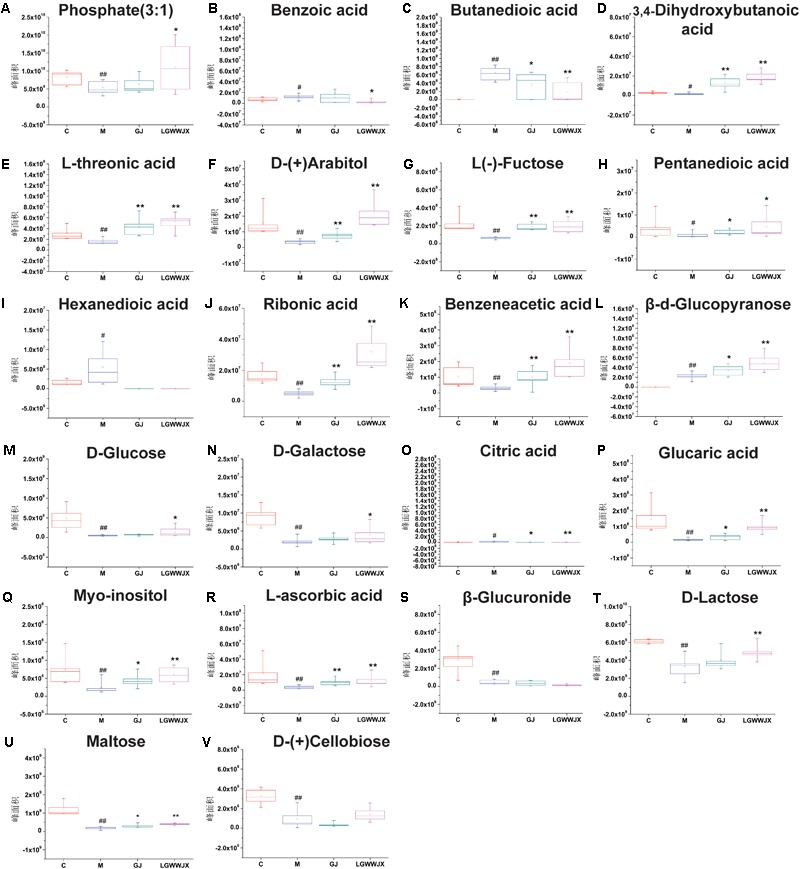
Levels differentially expressed metabolites in urine. **(A)** Phosphate(3:1); **(B)** Benzoic acid; **(C)** Butanedioic acid; **(D)** 3, 4-Dihydroxybutanoicacid; **(E)** L-threonic; **(F)** D-(+)arabitol; **(G)** L-(–)Fructose; **(H)** Pentanedioic acid; **(I)** Hexanedioic acid; **(J)** Ribonic acid; **(K)** Benzeneacetic acid; **(L)** β-D-Glucopyranose; **(M)** D-Glucose; **(N)** D-Galactose; **(O)** Citric acid; **(P)** Glucaric acid; **(Q)** Myoinositol; **(R)** L-Ascorbic acid; **(S)** β-Glucuronide; **(T)** D-Lactose; **(U)** Maltose; **(V)** D-(+)Cellobiose. C, control group; M, model group; GJ, Gan Jiang decoction group; LGWWJX, LGWWJX decoction group. ^#^*p* < 0.05, ^##^*p* < 0.01, compared with the control group. ^∗^*p* < 0.05, ^∗∗^*p* < 0.01, compared with the model group.

Biomarkers play an important role in the diagnosis and determination of prognosis of diseases. Based on the criteria VIP > 1, *p* < 0.05 and NIST match factor > 700, we identified 15 biomarkers in serum and 22 biomarkers in urine that were differentially present in the M group compared with the C group (Table 3). These potential biomarkers were mainly associated with carbohydrates, fatty acids, organic acids, and their products. In the M group, the majority of carbohydrates were significantly downregulated, while levels of D-(-)tagatose were increased and differences in glucopyranose in serum and urine were inconsistent (elevated in serum, reduced in urine), compared to the C group. Most fatty acid products were notedly upregulated in the M group, except for octadecanoic acid (serum), pentanedioic acid, and benzeneacetic acid (urine). Levels of propanoic acid, 3, 4-dihydroxybutanoic acid, and citric acid were also increased in the M group, while phosphate (3:1), glycerol, glycine, D-pinitol, and myoinositol were decreased in the M group compared to the C group. Intervention with GJ and LGWWJX decoctions reversed the observed changes for most of the differential metabolites, but they did not normalize glycerol (serum), hexanedioic acid, β-D-glucuronide, or D-(+)cellobiose (urine). All metabolites that were regulated by GJ were also regulated by the LGWWJX decoction. However, not all metabolites regulated by LGWWJX were regulated by GJ (Figure 5, 6 and Table 3). LGWWJX decoction normalized differential levels of glucopyranose (serum), benzoic acid, D-glucose, D-galactose, and D-lactose (urine), but GJ did not normalize these metabolites. In addition, LGWWJX decoction regulated serum D-glucose (serum and urine) and hexadecanoic acid, and urine phosphate (3:1), butanedioic acid, β-D-glucopyranose, citric acid, glucaric acid, myoinositol, and maltose.

**Table 3:** Potential biomarkers to distinguish control group rats, cold asthma rats, and rats treated with GJ and LGWWJX decoctions.

	RT/min	Endogenous metabolites	M/Z	VIP	*p*	Match	Regulation		
							C-M	M-GJ	M-LGWWJX
Sm1	5.333	Propanoic acid	28, 45, 73, 88, 117, 147, 191, 219	1.23	0.000	953	↑	↓	↓
Sm2	8.449	Glycerol	73, 103, 133, 147, 177, 205, 218	1.01	0.012	881	↓	/	/
Sm3	9.889	Glycine	45, 73, 86, 133, 147, 174, 188, 248, 276	1.72	0.023	824	↓	↑	↑
Sm4	11.115	Phosphate(3:1)	45, 73, 133, 211, 253, 283, 299, 314	1.43	0.015	776	↓	↑	↓
Sm5	19.798	Heptadecane	57, 71, 85, 99, 127, 155, 238	1.16	0.000	792	↑	↓	↓
Sm6	22.088	D-Pinitol	73, 147, 159, 247, 260, 318, 433	1.61	0.033	867	↓	↑	↑
Sm7	22.49	D-(-)-Tagatose	15, 45, 73, 103, 133, 147, 189, 217, 307, 466	1.96	0.000	874	↑	↑	↑
Sm8	22.634	D-Mannitol	73, 103, 147, 205, 217, 319, 345	1.18	0.000	858	↓	↑	↑
Sm9	22.853	D-Galactose	45, 59, 73, 103, 129, 147, 160, 205, 217, 229, 291, 319	1.26	0.000	922	↓	↑	↑
Sm10	23.069	D-Glucose	73, 103, 147, 205, 268, 319	1.40	0.015	922	↓	↑	↑
Sm11	24.363	Glucopyranose	73, 103, 129, 147, 191, 204, 217, 231	1.16	0.004	834	↓	/	↑
Sm12	25.131	Myoinositol	73, 103, 129, 147, 217, 305, 343, 432	1.14	0.000	891	↓	↑	↑
Sm13	27.786	Hexadecanoic acid	29, 43, 73, 83, 117, 132, 145, 159, 201, 243, 269, 313, 328	1.07	0.009	899	↑	↓	↓
Sm14	32.181	Octadecanoic acid	43, 55, 73, 117, 132, 145, 201, 243, 269, 341, 356	1.13	0.003	887	↓	↑	↑
Sm15	38.921	Lactose	73, 89, 103, 147, 204, 243, 271, 319	1.25	0.000	895	↓	↑	↑
Um1	10.938	Phosphate(3:1)	73, 115, 133, 211, 283, 299, 314	1.01	0.014	938	↓	/	↑
Um2	12.122 27.151	Benzoic acid	77, 105, 117, 147, 179, 194	1.15	0.021	923	↑	/	↓
Um3	12.932	Butanedioic acid	73, 129, 147, 172, 247	1.16	0.000	935	↑	↓	↓
Um4	14.120	3, 4-Dihydroxyb-utanoic acid	73, 147, 189, 233, 246, 300	1.13	0.021	914	↑	↑	↑
Um5	16.996	L-Threonic acid	73, 117, 147, 205, 292, 409	1.01	0.036	920	↓	↑	↑
Um6	19.630	D-(+)Arabitol	73, 103, 147, 205, 217, 243, 307, 395	1.08	0.009	913	↓	↑	↑
Um7	20.367, 20.509, 21.292	L(-)-Fructose	73, 89, 117, 133, 147, 201, 231	1. 02	0.040	867	↓	↑	↑
Um8	21.160	Pentanedioic acid	73, 112, 147, 186198, 229, 260, 288	1.06	0.009	850	↓	↑	↑
Um9	21.292	Hexanedioic acid	73, 109, 129, 147, 203, 233, 247, 363	1.21	0.009	774	↑	/	/
Um10	21.406	Ribonic acid	73, 103, 147, 217, 292, 307	1.09	0.002	909	↓	↑	↑
Um11	21.814	Benzeneacetic acid	73, 89, 105, 147, 164, 179, 252, 281	1.08	0.015	878	↓	↑	↑
Um12	22.380	β-D-Glucopyranose	73, 147, 191, 204, 291, 345, 435	1.42	0.000	928	↑	↑	↑
Um13	22.853	D-Glucose	73, 103, 147, 160, 205, 217, 319	1.01	0.002	923	↓	/	↑
Um14	22.862	D-Galactose	73, 147, 160, 205, 217, 229, 291, 319	1.18	0.000	930	↓	/	↑
Um15	23.420	Citric acid	73, 147, 183, 211, 231, 273, 285, 363, 465	1.01	0.006	910	↑	↓	↓
Um16	23.958	Glucaric acid	73, 103, 147, 292, 333, 423	1.00	0.010	708	↓	↑	↑
Um17	25.138	Myo-inositol	73, 103, 147, 265, 305, 367, 432	1.001	0.012	879	↓	↑	↑
Um18	25.523	L-Ascorbic acid	73, 133, 147, 205, 243, 274, 361, 391	1.08	0.042	800	↓	↑	↑
Um19	38.591	β-Glucuronide	73, 103, 147, 180, 257, 375	1.07	0.000	923	↓	/	/
Um20	38.821	D-Lactose	73, 103, 129, 147, 204, 217, 319, 361, 392	1.147	0.000	928	↓	/	↑
Um21	39.434	Maltose	73, 147, 204, 217, 361, 373, 392	1.19	0.000	824	↓	↑	↑
Um22	40.406	D-(+)Cellobiose	73, 103, 147, 191, 204, 217, 243, 361, 373, 393	1.17	0.000	928	↓	/	/

*↑ Represents content increased; ↓ represents content decreased.*

### Metabolic Pathway Analysis

**FIGURE 7:**
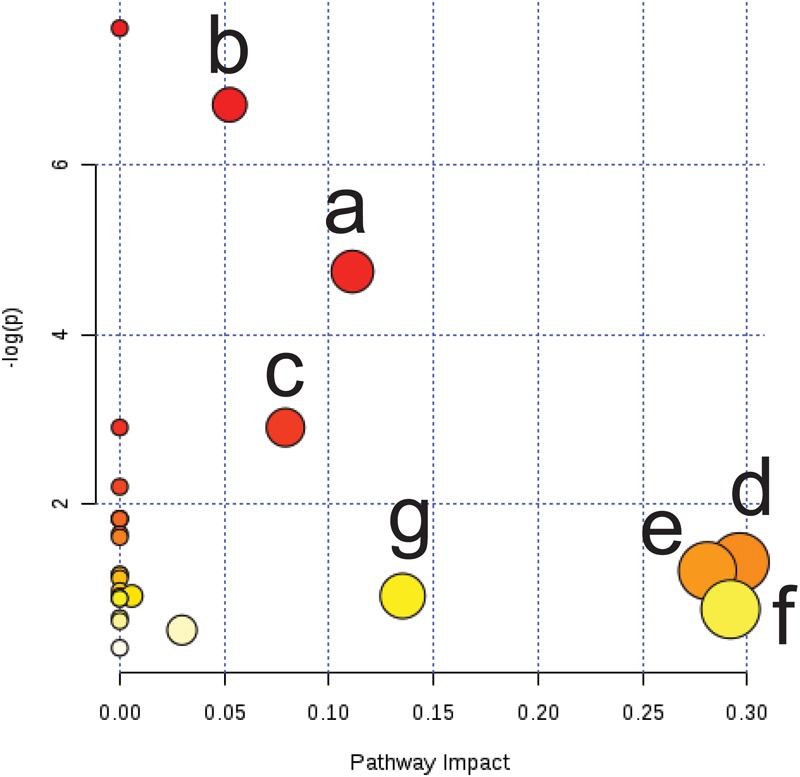
Summary of pathway analysis of serum and urine samples from cold asthma rats. **(a)** Starch and sucrose metabolism; **(b)** Galactose metabolism; **(c)** Tricarboxylic acid cycle; **(d)** Glyoxylate and dicarboxylate; **(e)** Glycerol phospholipid metabolism; **(f)** Glycine, serine, and threonine metabolism; **(g)** Inositol phosphate metabolism.

To explore the possible pathways affected by cold asthma, differential endogenous metabolites in Table 3 were imported into MetaboAnalyst4.0^2^. The impact value threshold of pathway topology analysis was set to 0.1, and the impact value of pathways above this threshold were considered potential target pathways. As shown in Figure 7, cold asthma was associated with seven key metabolic pathways, including starch and sucrose metabolism, galactose metabolism, the tricarboxylic acid cycle (TCA), glyoxylate and dicarboxylate, glycerol phospholipid metabolism, glycine, serine and threonine metabolism, and inositol phosphate metabolism.

### Correlation Between Metabolites and Various Efficacy Indicators

**FIGURE 8:**
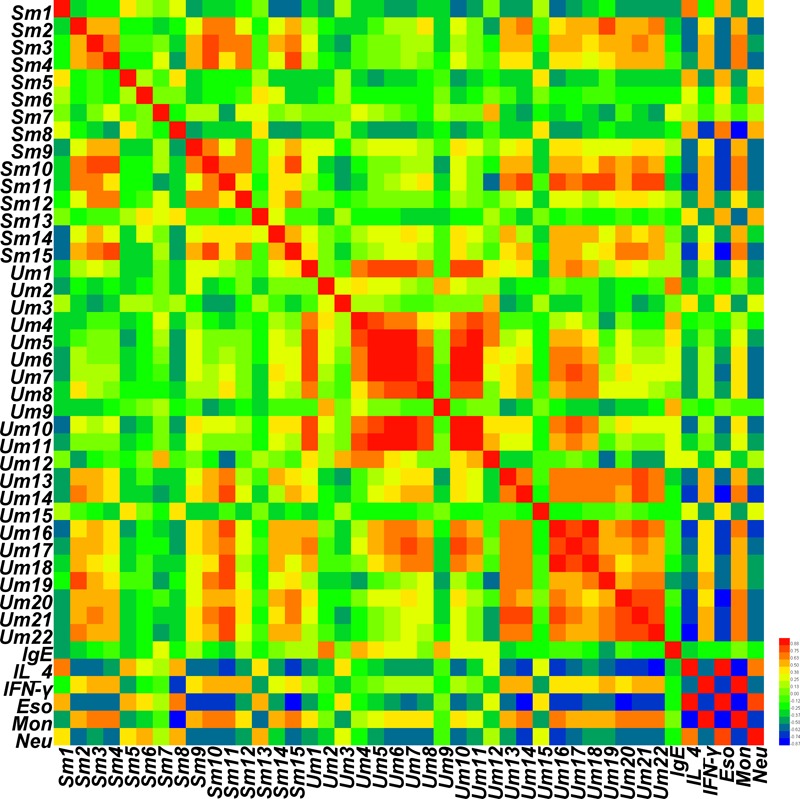
Correlation map of rat serum and urine metabolites and pharmacodynamics indices according to Pearson correlation coefficients. Degrees of correlation degrees are shown using a color scale from significantly negatively correlated (blue) to significantly positively correlated (red).

A correlation map of rat serum and urine metabolites and efficacy indicators of cold asthma was generated based on Pearson’s correlation coefficients. The correlation heat-map (Figure 8) shows that the metabolites of Sm1, Sm5, Sm8 (serum; propanoic acid; heptadecane) were strongly positively related to the level of IL-4, eosinophils, and neutrophils (*r* = 0.645, 0.599, 0.493; 0.554, 0.582, 0.422; 0.558, 0.647, 0.563, respectively). In addition, Sm8 was also negatively associated with IFN-γ (*r* = -0.733). However, the metabolites Sm2, Sm3, Sm4, Sm9, Sm10, Sm11, Sm15 (serum glycerol, glycine, phosphate, D-galactose, glucose, glucopyranose, and lactose) were positively associated with IFN-γ. The metabolites Um2, Um4, and Um9 (urine benzoic acid, 3,4-dihydroxybutanoic acid, and hexanedioic acid) were positive associated with IgE (0.722, 0.605, 0.571). The metabolites Um13, Um14, Um16, Um20, Um21, and Um22 (urine D-glucose, D-galactose, glucaric acid, D-lactose, maltose, and D-(+)cellobiose) were negatively associated with IL-4, eosinophils, and neutrophils (-0.542, -0.584, -0.412; -0.669, -0.774, -0.671; -0.659, -0.707, -0.638; -0.642, -0.745, -0.607; -0.700, -0.741, -0.583; -0.743, -0.729, -0.568), and positively associated with IFN-γ (0.526; 0.538; 0.428; 0.460; 0.584; 0.522).

## Discussion

This study evaluated the protective effects of GJ and LGWWJX on cold asthma induced by OVA challenge and swimming in ice-water in rats at the metabolomics level. Seven metabolic pathways were affected in cold asthma. Based on the identified potential biomarkers and associated metabolic pathways, the observed changes were associated with energy metabolism and oxidative stress in cold asthma rats. GJ and LGWWJX reversed the majority of metabolite changes that resulted from cold asthma. LGWWJX exerted more powerful beneficial effects than GJ on cold asthma.

### Energy Metabolism

In this study, a prominent metabolic feature observed in cold asthmatic rats was a change in energy metabolism. Three major sugar metabolic pathways are involved in energy metabolism: starch and sucrose metabolism, galactose metabolism, and the TCA cycle. Carbohydrates, proteins, and fats are the main sources of energy in mammals. Previous studies showed that energy metabolism markers were reduced in urine and serum in models of experimental asthma (Ho et al., 2013; Hui et al., 2017). Carbohydrates are generally catabolized in cells via the concerted action of glycolysis and the TCA cycle under aerobic condition (Saude et al., 2009). Carbohydrates are converted to propanoic acid by pentose phosphate under anoxic conditions. In our study, elevated propanoic acid (a product of glycolysis), citric acid (a product of the TCA cycle), and butanedioic acid (succinic acid, product of the TCA cycle) demonstrated alternations in energy metabolism. These products typically accumulate during abnormal lung respiration, especially under hypoxic or inflamed conditions (Wolak et al., 2009). Increased propanoic acid levels have been reported in the serum of adults with asthma, in the urine of children with asthma, and in BALF from patients with cystic fibrosis (Jung et al., 2013; Alvarez et al., 2017; Quan-Jun et al., 2017). Citric acid is a metabolic product carbohydrate breakdown via the TCA cycle that provides additional energy in the form of adenosine triphosphate (ATP). Increased citric acid has been reported in the plasma of adults with cystic fibrosis during pulmonary crises and in the urine of asthmatic rats, which demonstrated consistent impairment of energy metabolism in these respiratory diseases (Peng et al., 2014). Elevated levels of butanedioic acid (succinic acid) were observed in the serum of patients with moderate asthma, and in the urine of children with asthma (Jung et al., 2013; Peng et al., 2014; Chang et al., 2015). In addition, we found that levels of 3,4-dihydroxybutanoic acid were also elevated in the M group, which could indicate energy metabolism disorders related to the increased ketone bodies (β-oxidation products of free fatty acids), which can provide energy to organs such as the brain when carbohydrate stores are depleted (Wu et al., 2018). In addition, significant correlations have been observed between energy metabolism metabolites and inflammatory cells in asthma, supporting a relationship between energy metabolism and inflammation. In this study, carbohydrate-related metabolites such as D-galactose, D-glucose, glucopyranose, D-mannitol, lactose, and other metabolites were decreased in the model group rats compared with the control group rats. These sugar and sugar alcohols might have been broken down into propanoic acid, citric acid, and butanedioic acid to provide additional energy, consistent with results from a previous study (Hui et al., 2017). Changes in energy metabolism were the main characteristics in this study, which may have been related to increased respiratory burden, and increased use of energy to recruit inflammatory cells and support increased energy demands to maintain body temperature in cold asthma rats. GJ and LGWWJX decoctions normalized the majority of metabolites altered in the cold asthma model, and LGWWJX exerted more powerful effects than GJ on energy metabolism. This difference might have been due to the change of property of warming and the complex chemical composition under the compatibility of GJ in LGWWJX. Cui et al. (2018) showed that the active components in dried ginger for treatment of cold-related lung fluid retention in rats with COPD have different tissue distribution.

### Oxidative Stress

Oxidative stress is closely associated with asthma (Sedgwick et al., 1990; Vachier et al., 1992; Dworski, 2000). Inflammatory cells such as eosinophils and neutrophils release reactive oxygen species (ROS) in asthmatic patients (Fitzpatrick et al., 2014). Reduction in antioxidant capacity and overproduction of ROS results in redox imbalance. ROS contribute to the pathogenesis of asthma through pulmonary function impairment, mast cell degranulation, airway remodeling, and mucus secretion by epithelium, which results in aggravation of local inflammation the lung (Zhang et al., 2018). In this study, three pathways may have been involved in cold asthma-related oxidative stress: lipid peroxidation, glycine, serine, and threonine metabolism, and inositol phosphate metabolism.

### Lipid Peroxidation

Lipid peroxidation is a consequence of oxidative stress characterized by degradation of polyunsaturated fatty acids into volatile organic acids (VOCs), such as benzoic acid, glutaric acid, and heptaric acid, alkanes, and aldehydes, which are exlahed via the lungs (Cap, 2004; Fehrenbach et al., 2014; Zhang et al., 2018). Loureiro et al. (2016) examined the relationship between oxidative stress, eosinophilic inflammation, and disease severity in asthmatic patients through a metabolomics study of alkanes and aldehydes. This study demonstrated that increased alkanes and aldehydes positively correlated with asthma severity and eosinophils. Increased benzoic acid, hexanedioic acid, (urine), heptadecane, and hexadecenoic acid (serum), and decreased octadecanoic acid (serum), pentanedioic acid, and benzeneacetic acid (urine) in the M group in our study indicated abnormal lipid peroxidation, which may have been related to oxidative stress in cold asthma. Increased levels alkanes and aldehydes were also reported to be related to severity of asthma (Loureiro et al., 2014). In addition, a significant correlation was observed between heptadecane, benzoic acid, and hexanedioic acid, and inflammatory cells in asthma, which suggested that oxidative stress may play a role in the pathogenesis of cold asthma. GJ and LGWWJX reversed these changes, and the effect of LGWWJX decoction was more pronounced than that of GJ.

### The Glycine, Serine, and Threonine Metabolism Pathway

Glycine, serine, and threonine are closely related amino acids that share common biochemical pathways. Glycine can be converted to serine, then choline and cholamine, which participate in the biosynthesis of purines and pyrimidines. Wang et al. (2014) studied the cytoprotective effects of glycine and indicated that glycine inhibits oxidative stress through enhancing intracellular glutathione concentrations in IPEC-1 cells. Legendre et al. (2009) found that glycine can reduce inflammatory mediators and ROS. Grotz et al. (2001) showed that glycine can significantly reduce lung injury by inhibiting inflammatory cell aggregation and attenuating lipid peroxidation. In our study, glycine levels were decreased in the serum of cold asthma rats and the glycine, serine, and threonine metabolic pathway was disrupted. In addition, glycine, phosphate (3:1), glycerol, and myoinositol are raw materials for synthesis of phospholipids, which are important components of cell membranes. Reduced levels of these metabolites reflected decreased phospholipid synthesis. We speculated that reduced glycine in cold asthma rats may have been related to oxidative stress and inflammatory cell infiltration, but the specific mechanisms have not been characterized.

### Inositol Phosphate Metabolism

Altered levels of myoinositol are related to inositol phosphate metabolism. Phosphatidylinositol 3-kinases (PI3K), a key element in inositol phosphate metabolism, generates lipid second messengers that control an array of intracellular signaling pathways which play important roles in inflammation. A previous study showed that oxidative stress is associated with the PI3K signaling pathway. Ng et al. (2014) showed that artesunate can suppress cigarette smoke-induced airway inflammation and oxidative damage in mice, likely via inhibition of the PI3K signaling pathway. Rossios et al. (2012) showed that formoterol reversed oxidative stress-induced corticosteroid insensitivity and decreased β2 adrenoceptor-dependent cAMP production via inhibition of PI3K signaling, which suggested a relationship between oxidative stress and the PI3K pathway. Other factors related to inositol phosphate metabolism are also strongly associated with asthma. Mizuta et al. (2015) showed that long-chain free fatty acid binding to fatty acid receptors increased inositol phosphate synthesis in human airway smooth muscle cells (hASM). Brown et al. (2016) showed that flavonols significantly inhibited purified phosphodiesterase-4 (PDE4) and PLCβ enzymes, attenuated acetylcholine-induced increases in inositol phosphates and CPI-17 phosphorylation in hASM cells and relaxed airway smooth muscle. These studies indicated the crucial role of inositol phosphate metabolism in asthma. In our study, decreased myoinositol and pathway analysis suggested disruption of inositol phosphate metabolism was a characteristic of cold asthma, and GJ and LGWWJX both normalized myoinositol levels. However, the specific mechanisms of these effects have not been characterized.

### Other Metabolites

In addition to metabolites that contribute to energy metabolism and oxidative stress, other metabolites were associated with cold asthma. L-Ascorbic acid (vitamin C) is an important low molecular weight antioxidant that maintains antioxidative defenses at the air-lung interface (Nirina et al., 2015). Previous studies have shown that lower levels of essential antioxidants in circulation were associated with increased risk of disease (Manju et al., 2002; Saygili et al., 2002; Aydin et al., 2006). Absorption of ascorbic acid in the small intestine occurs primarily through Na^+^-dependent active transport (Rahman et al., 2017). In this study, reduced L-ascorbic acid suggested alterations in the antioxidant defense system, which can result in oxidative stress. Previous reports showed that vitamin C can reduce histamine, which results in mast cell degranulation allergic reactions, but the mechanisms that result in these allergic effects have not been characterized (Hagel et al., 2013; Romero et al., 2015). Vollbracht et al. (2018) observed ascorbate deficiency in patients with allergic diseases and treatment with intravenous high-dose vitamin C reduced allergy-related symptoms. In addition, in our study, the level of D-pinitol was also decreased. D-pinitol exerts insulin-like effects via facilitation of transport of creatine and other nutrients into muscle cells (Davis et al., 2000). D-Pinitol also exhibits anti-inflammatory activity and can contribute to prevention of cardiovascular diseases (Singh et al., 2001; Kim et al., 2005). Lee et al. (2007) showed that D-pinitol reduces allergic airway inflammation and hyperresponsiveness due to alteration of Th1/Th2 polarization via suppression of GATA-3 and increased T-bet expression. In our study, GJ and LGWWJX decoctions normalized L-ascorbic acid and D-pinitol in M group rats, but the mechanisms were not clear.

### Cold Asthma

Cold weather affects the respiratory epithelium and induces bronchial hyperresponsiveness (Liener et al., 2003; Cruz and Togias, 2008). Cold weather further aggravates respiratory symptoms in asthma (Hyrkäs-Palmu et al., 2018). Cold asthma is a syndrome diagnosed in CM clinics. CM doctors postulate that cold will injure the spleen, and chronic spleen injury will increase sputum drinking, which accumulates in the lung, resulting in asthma. As such, CM doctors advocate for “warming the stomach to dispel cold, warming the lung to resolve fluid” to treat cold asthma. GJ has warm and spicy properties and it is often used in combination with other herbs to treat cold asthma in clinics. LGWWJX, which contains GJ, is the main prescription for treating cold asthma. In this prescription, GJ is the main component and it can warm the lung and spleen. Asari Radix Et Rhizoma helps GJ to warm the lung, Poria helps GJ tonify the spleen and dissipate excessive fluid, Schisandrae Chinensis Fructus astringes the lung to stop cough, and Glycyrrhizare Radix Et Rhizoma moderates the properties of the herbs.

It is generally accepted that the effects of CM prescriptions are closely related to their chemical compositions. As such, modern research has also focused on chemical characterization of Chinese remedies. Gingerols, major medicinal ingredients in dried ginger, exert antipyretic, analgesic, anti-inflammatory, and antioxidant effects (Bak et al., 2012; Townsend et al., 2014; Liang et al., 2018). A previous study reported that drying temperature of ginger results in conversion of gingerols to shogaols, resulting in increased antioxidant and antimicrobial activities (Ghasemzadeh et al., 2018). In our previous study, we found that other herbs in LGWWJX decoction combined with dried ginger affected the concentrations of 6-gingerol, 8-gingerol, 6-shogao, and 10-gingerol (Ran et al., 2018). However, it is difficult to characterize the interactions between GJ and other herbs in LGWWJX decoction, and to determine which components have the greatest effects on cold asthma. However, the advantages of metabolomics were apparent in this study.

## Conclusion

In this study, serum and urine metabolites identified by GC–MS and general pharmacodynamic evaluation (morphological observation, histopathology, inflammatory factors, and visceral indices) were used to compare the effects of GJ and LGWWJX decoctions on cold asthma. Significant metabolic abnormalities were observed for 37 metabolites (15 in serum, 22 in urine) in rats with cold asthma. These altered metabolites might be potential biomarkers or therapeutic targets during development of cold asthma. Furthermore, pathway analysis demonstrated that pathways associated with energy metabolism and oxidative stress were disrupted in rats with cold asthma. GJ and LGWWJX regulated metabolites associated with various pathways. We demonstrated that the CM prescription LGWWJX exerted stronger effects than the single herb, GJ, at the metabolite level. However, the specific mechanisms of compatibility of these Chinese Medicines requires further study.

## Author Contributions

YQH, XW, and YH conceived and designed the experiments. SR, FS, and YS performed the experiments. SR and YQH drafted this manuscript and analyzed the data. YH and XW provided final approval of the version to be published. All of the authors discussed the complete dataset to establish an integral and coherent analysis.

## Conflict of Interest Statement

The authors declare that the research was conducted in the absence of any commercial or financial relationships that could be construed as a potential conflict of interest.

## Footnotes

1 https://xcmsonline.scripps.edu

2 http://www.metaboanalyst.ca/MetaboAnalyst/

## Supplementary Material

FIGURE S1 UPLC-Q-TOF/MS Total ion Chromatogram of LGWWJX Decoction.

FIGURE S2 BALF inflammatory cells and differential counting in each group.

FIGURE S3 Serum inflammatory cells of rats in each group.

FIGURE S4 GC-MS TIC chromatograms of QC samples in rats serum (A) and urine (B).

Table S1 Characterization of chemical composition in LGWWJX decoction by UPLC-Q-TOF/MS.
