# Supplementary figures and images for: The Study of Dried Ginger and Linggan Wuwei Jiangxin Decoction Treatment of Cold Asthma Rats Using GC–MS Based Metabolomics
Source: Front Pharmacol. 2019 Apr 11;10:284. doi: 10.3389/fphar.2019.00284 (PMC6470627; doi:10.3389/fphar.2019.00284)

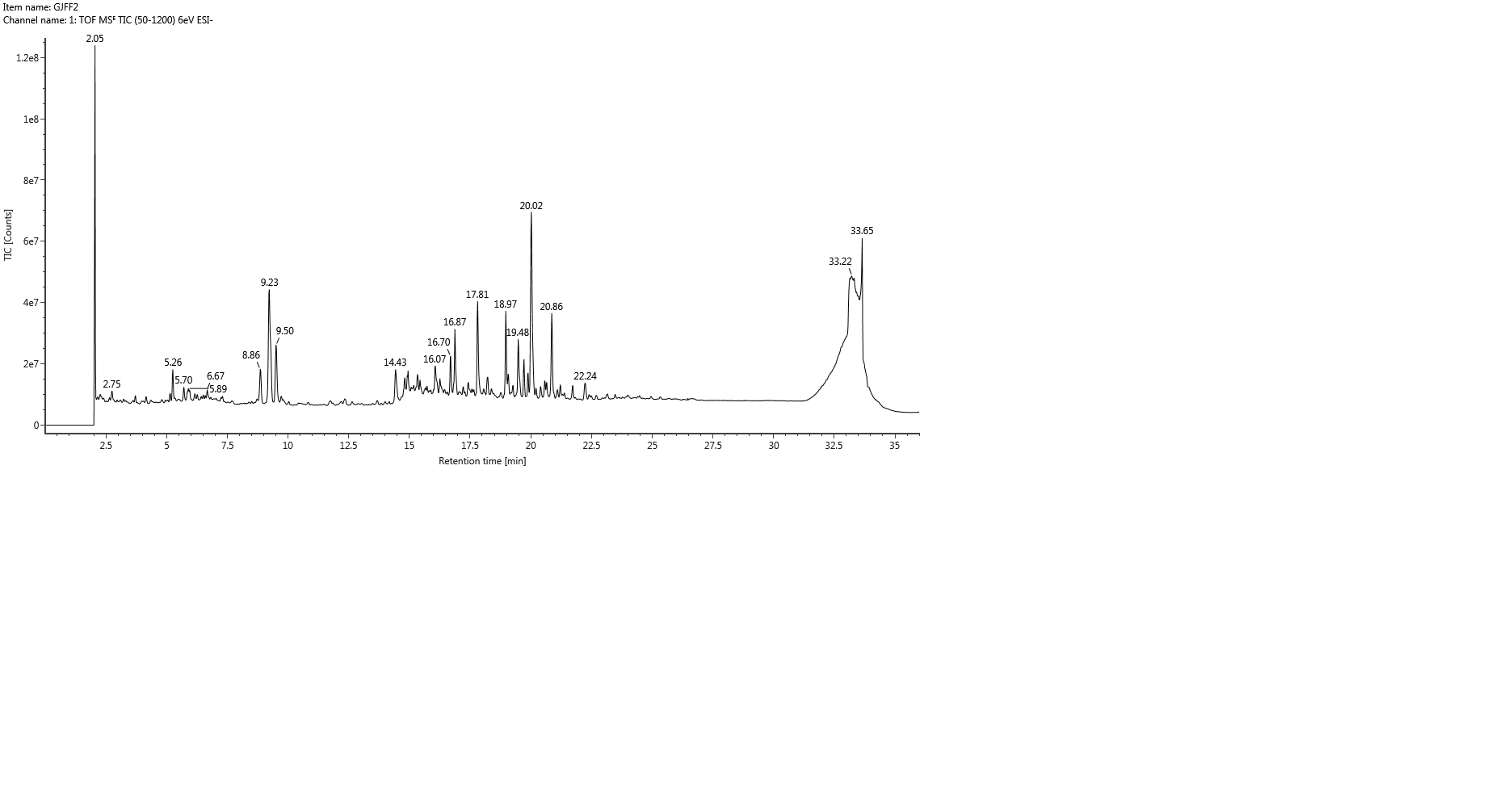

Supplement: FIGURE S1 — UPLC-Q-TOF/MS Total ion Chromatogram of LGWWJX Decoction. [file Image_1.TIF]

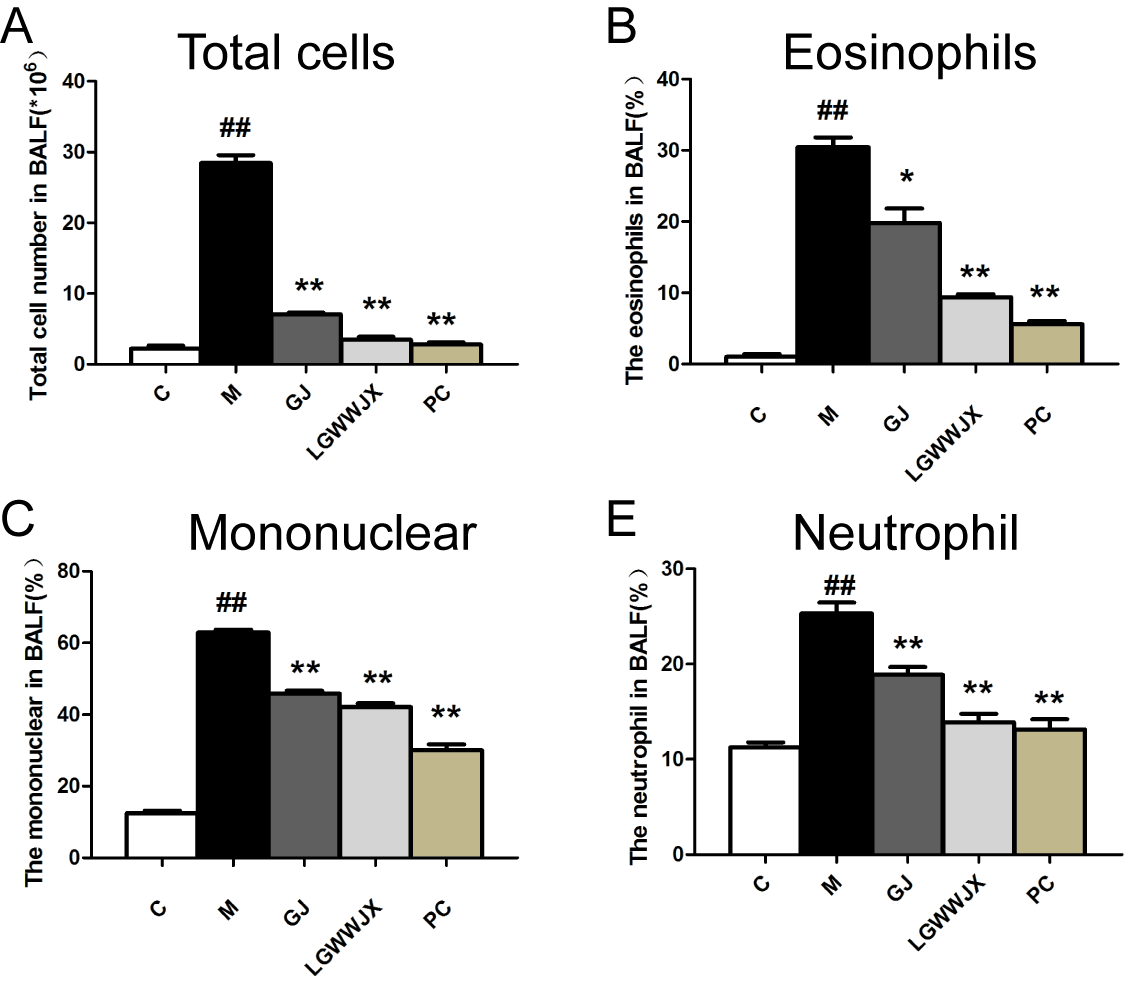

Supplement: FIGURE S2 — BALF inflammatory cells and differential counting in each group. [file Image_2.TIF]

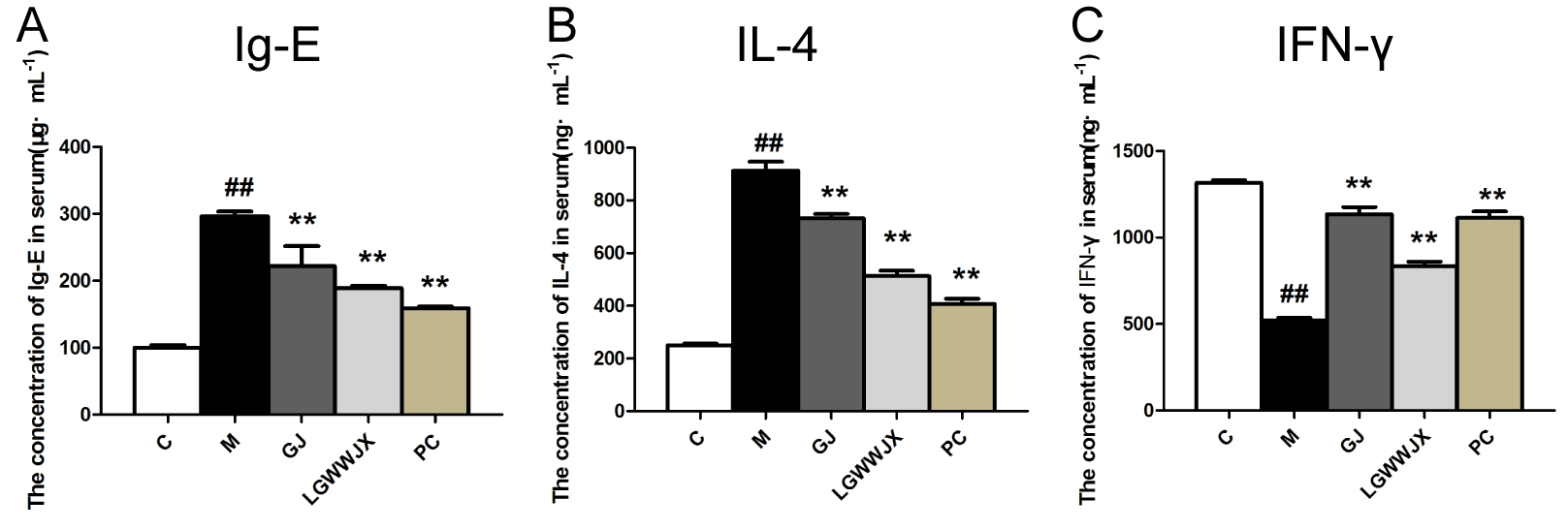

Supplement: FIGURE S3 — Serum inflammatory cells of rats in each group. [file Image_3.TIF]

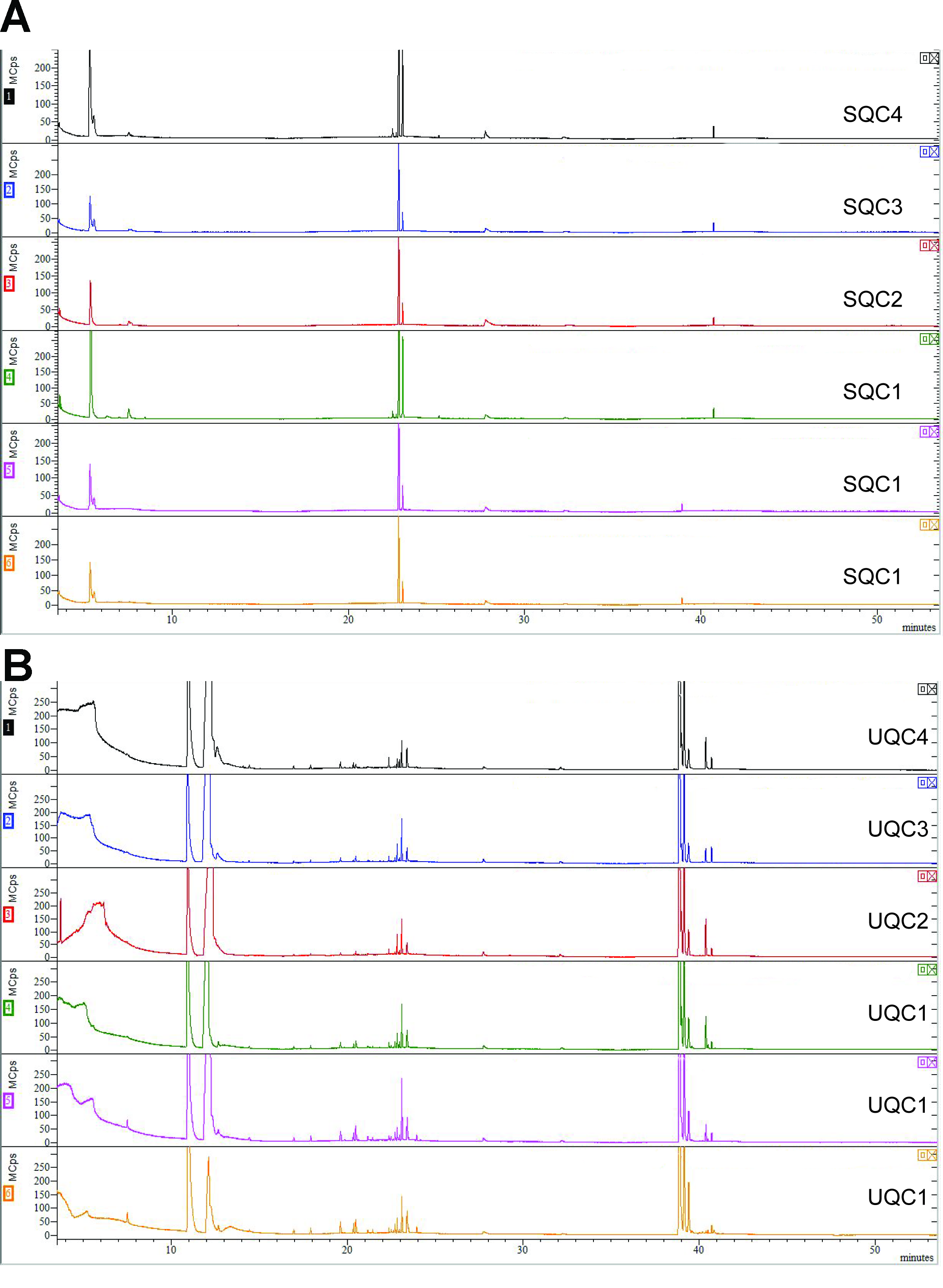

Supplement: FIGURE S4 — GC-MS TIC chromatograms of QC samples in rats serum (A) and urine (B). [file Image_4.TIF]
