# Supplementary material for: The Study of Dried Ginger and Linggan Wuwei Jiangxin Decoction Treatment of Cold Asthma Rats Using GC–MS Based Metabolomics
Source: Front Pharmacol. 2019 Apr 11;10:284. doi: 10.3389/fphar.2019.00284 (PMC6470627; doi:10.3389/fphar.2019.00284)
Supplement: Table S1 — Characterization of chemical composition in LGWWJX decoction by UPLC-Q-TOF/MS. [file Table_1.DOCX]

Table 1 Characterization of chemical composition in LGWWJX decoction by LC-QTOF-MS/MS

| No | r(min) | m/z | Formula | Compound name |
| --- | --- | --- | --- | --- |
| 1 | 5.27 | 166.0634 | C9H10O3 | veratraldehyde |
| 2 | 8.85 | 578.16356 | C27H30O14 | isoviolanthin |
| 3 | 9.29 | 433.1140 | C21H22O10 | Cheorospondin |
| 4 | 9.54 | 418.1269 | C21H22O9 | Isoliquiritin |
| 5 | 16.89 | 498.2256 | C28H34O8 | Angeloylgomisin O |
| 6 | 18.99 | 372.1572 | C21H24O6 | Hydroxy-1,7-bis  (4-hydroxy-3-methoxyphenyl)-3-heptanone |
| 7 | 20.03 | 821.3977 | C42H62O16 | Glycyrrhizin |
| 8 | 22.24 | 294.1837 | C17H26O4 | 6-gingerol |
